# Composite hepatocellular carcinoma and small cell carcinoma with early nodal metastasis: A case report

**DOI:** 10.1097/MD.0000000000007868

**Published:** 2017-08-25

**Authors:** Yu-Jen Liu, Kwai-Fong Ng, Shih-Chiang Huang, Ren-Chin Wu, Tse-Ching Chen

**Affiliations:** a Department of Pathology, Chang Gung Memorial Hospital, Guishan; b Department of Pathology, Chang Gung University School of Medicine, Taoyuan, Taiwan.

**Keywords:** composite tumor, hepatocellular carcinoma, neuroendocrine carcinoma, neuroendocrine tumor, nodal metastasis, small cell carcinoma

## Abstract

**Rationale:**

Hepatocellular carcinoma (HCC) is known to grow in a mosaic pattern, and it can sometimes be combined with non-hepatocellular cells. Despites the variety of combination, HCC with a significant neuroendocrine carcinoma (NEC) component remains very rare. Most of the reported cases were treated as conventional HCC with a relatively poor prognosis. Early diagnosis may lead to a better treatment modality. Here, we report a case of composite HCC and small cell carcinoma (SCC) with nodal metastasis of the SCC component alone.

**Patient concerns:**

A 65-year-old man with chronic viral hepatitis C presented with abdominal discomfort for 2 months. Computed tomography and angiography of the liver showed a 4.3 cm hypervascular tumor in segment 4 and enlargement of the perihilar and paracaval lymph nodes.

**Interventions:**

Extended left lobectomy and regional lymph node dissection were performed.

**Diagnosis:**

The hepatic tumor was heterogeneous with two distinct gross components. The green part showed a grade III hepatocellular carcinoma with an immunoreaction to Hep Par 1, glypican 3 and α-fetoprotein, whereas the white part exhibited a small cell carcinoma, as evidenced by expressions of chromogranin A and synaptophysin. The lymph node was metastasized by the SCC component. The SCC part was also positive for vimentin with perivascular accentuation. ß-catenin immunostain showed reduced membranous expression in the SCC component, as compared to HCC.

**Outcomes:**

The patient expired 39 days after the surgical intervention.

**Lessons:**

Clinicians should be highly alert to a composite hepatic tumor, especially in dealing with a small heterogeneous tumor (< 5 cm) with early lymph node metastasis.

## 1 Introduction

Hepatocellular carcinoma (HCC) is the most common tumor in the liver. It is composed of tumor cells with hepatocellular differentiation, arranged in a trabecular, acinar, or compact pattern. Cytological variants include pleomorphic cells, clear cell, spindle cell, and oncocytic cells with or without fatty change, hyaline bodies, pale bodies or ground glass inclusion, and bile secretion.^[1]^ HCC often grows in a mosaic pattern, in which different cell types arrange in different architectural patterns in a large tumor. HCC can be occasionally combined with other cell types with nonhepatocellular differentiation. Combined hepatocellular-cholangiocarcinoma is the most common combination.^[2]^

HCC with a significant neuroendocrine carcinoma (NEC) component is rare. Only 17 cases have been reported in English literature.^[3–15]^ Most of these patients were treated as conventional HCC. Their prognosis was relatively poor. Here, we present a case of collision HCC and small cell carcinoma (SCC) with early nodal metastases by pure SCC. The morphological spectrum, immunophenotypes, and the clinical significance of the NEC component are discussed.

## 2 Case presentation

A 65-year-old Taiwanese man, a carrier of viral hepatitis C without previous medical intervention, suffered from abdominal discomfort for 2 months. Abdominal ultrasonography revealed a mass in segment 4 of the liver. Biochemical data are summarized in Table 1. The chest radiograph was unremarkable. Computed tomography and angiography of the liver showed a hypervascular hepatic tumor in segment 4 and enlargement of the perihilar and paracaval lymph nodes. No lesion was identified in the pulmonary, gastrointestinal, or genitourinary system on the chest to pelvis computed tomography study. He underwent extended left lobectomy and regional lymph node dissection. Neither portal vein thrombus nor extrahepatic involvement was identified intraoperatively. Frozen sections of group 8 and 12 lymph nodes showed metastatic SCC.

**Table 1:**
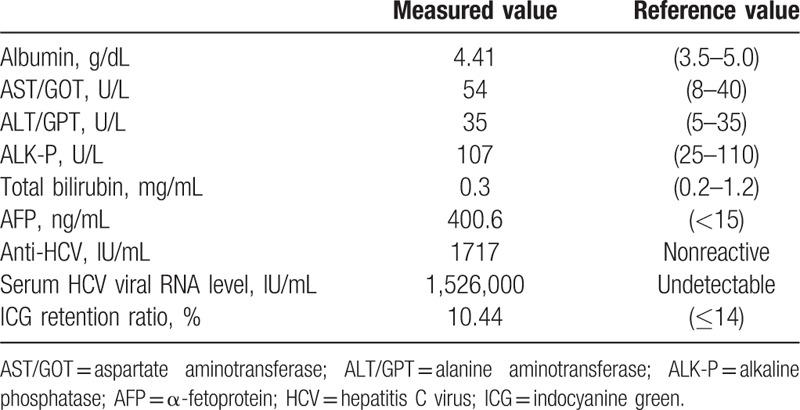
Laboratory data of present case.

## 3 Methods

The lymph node specimen was fixed with 10% buffered formalin after intraoperative frozen consultation, and so was the resected liver specimen. All specimens were then embedded in paraffin, cut into 4-μm-thick sections, and stained with hematoxylin and eosin. An immunohistochemical study was performed on selected blocks. The panel of antibodies used is listed as follows: α-fetoprotein (clone NCL-AFPp; 1:50; Novocastra), Hep Par 1 (clone OCH1E5; 1:200; DAKO), glypican 3 (clone 1G12; 1:300; Cellmarque), vimentin (clone V9; 1:1000; DAKO), synaptophysin (clone 27G12; 1:400; Novocastra), chromogranin A (clone DAK-A3; 1:500; DAKO), β-catenin (clone 17C2; 1:100; Novocastra), and Ki-67 (clone MIB-1; 1:100; DAKO). The study was approved by Institutional Review Board of Chang Gung Medical foundation (101-4589A3).

## 4 Results

### 4.1 Histopathological findings

**Figure 1:**
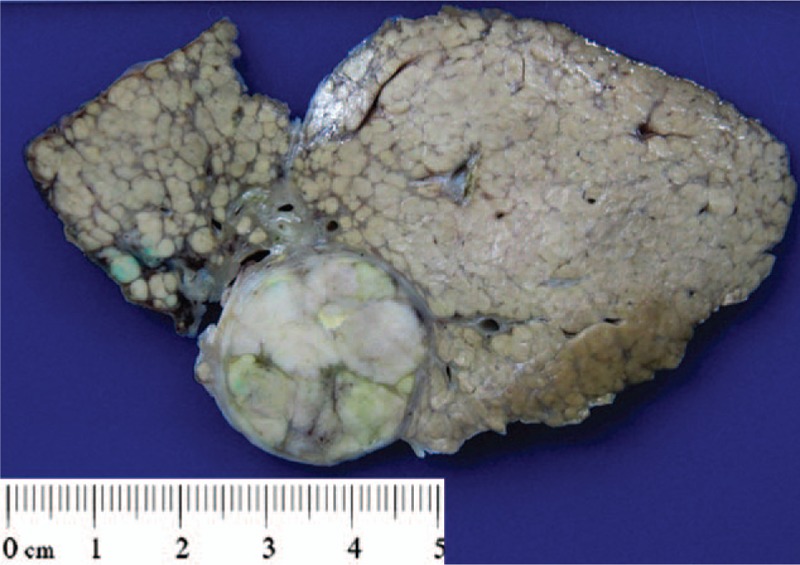
Gross picture of the resected liver. A 4.3 cm partially encapsulated tumor exhibits solid, green, and focally tan white cut surface. The background liver shows diffuse nodularity.

**Figure 2:**
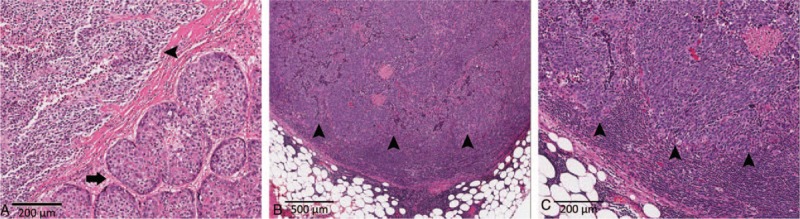
Histology of the liver tumor. A, The liver tumor is composed of mixed hepatocellular carcinoma (HCC, arrow) and small cell carcinoma (SCC, arrowhead) components. The 2 components are separated by fibrous bands (*original magnification ×100*). B and C, The metastasized lymph nodes harbor only the SCC component (arrowhead) (*Original magnification ×40 and ×100*, respectively).

The resected liver showed diffuse nodularity. The hepatic tumor measured 4.3 × 3.9 × 2.4 cm. It was partially encapsulated, green, solid, and focally tan white (Fig. 1). Microscopically, the hepatic tumor showed 2 distinct components. The tumor cells in the green area were large and polygonal, with trabecular arrangement, abundant eosinophilic cytoplasm, and hyperchromatic nuclei (Fig. 2A). This component was a classical grade III HCC. The grossly white-to-tan component was composed of solid sheets of small tumor cells, displaying scant cytoplasm, salt-and-pepper nuclei, stipple chromatin, geographic necrosis, and frequent mitotic figures (>30/10 HPF). This component morphologically fulfilled the criteria of SCC (Fig. 2A). No transition zone was found between HCC and SCC.

### 4.2 Immunohistochemical findings

**Figure 3:**
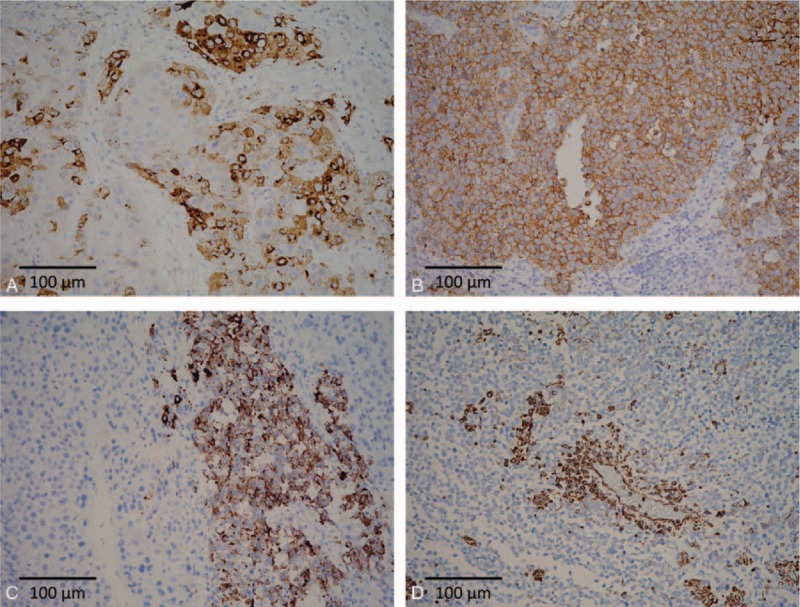
Immunophenotypes of the composite tumor. A, The HCC component is immunopositive for α-fetoprotein. B and C, The SCC component is immunoreactive to both synaptophysin (B) and chromogranin A (C). D, The tumor cells in SCC component are immunoreactive to vimentin with perivascular accentuation (*original magnification ×200*).

**Figure 4:**
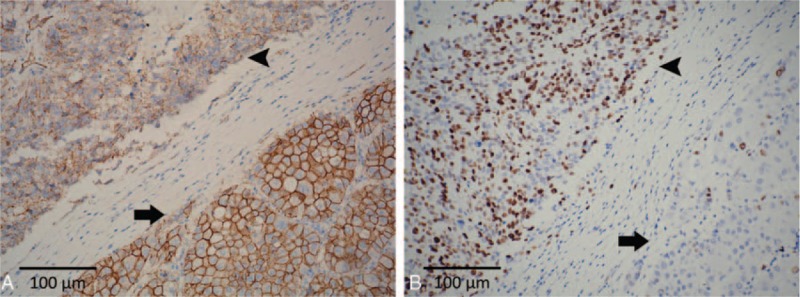
A, β-catenin staining demonstrates strong membranous expression in the HCC component (arrow), and reduced membranous staining in the SCC component without cytoplasmic and nuclear staining (arrowhead). B, The Ki-67 labeling index is remarkably higher in the SCC component (arrowhead) than in the HCC component (arrow) (*original magnification ×200*).

The HCC component was positive for α-fetoprotein (Fig. 3A), Hep Par 1, and glypican 3 and negative for vimentin, synaptophysin, and chromogranin A. The SCC component was positive for synaptophysin, chromogranin A, and vimentin (Fig. 3B–D). The SCC component showed reduced membranous staining (Fig. 4A), in contrast to the strong membranous expression of β-catenin in the HCC component (Fig. 4A). The Ki-67 labeling index of the SCC component was >80% (Fig. 4B). The group 8 and group 12 lymph nodes were largely effaced by the metastatic nests of SCC (Fig. 2B and C), with identical immunophenotype to its hepatic primary.

### 4.3 Final diagnosis

The final pathology of the resected specimen reported a mixed SCC and HCC. It was classified as pT2N1M0, stage 4a, according to the American Joint Cancer Committee 7th edition staging system. The patient died of rapidly deteriorating liver and renal functions 39 days after surgery.

## 5 Discussion

Primary mixed HCC and NEC is very rare. In 1235 hepatic tumors investigated by Nomura et al,^[14]^ the incidence was 0.46%. Including our patient, there are currently 18 reported cases of HCC with an NEC component in English literature.^[3–15]^ All cases were associated with either viral hepatitis B or C except for the case described by Baker et al.^[3]^ All cases were men and the mean age was 64.6 (range: 43–76). Eleven of the cases were treated with surgery alone (including 1 radiofrequency ablation), 1 with chemotherapy alone, and 6 with surgery followed by transarterial chemoembolization or chemotherapy. The clinicopathological profiles of the 18 cases are summarized in Table 2.

**Table 2:**
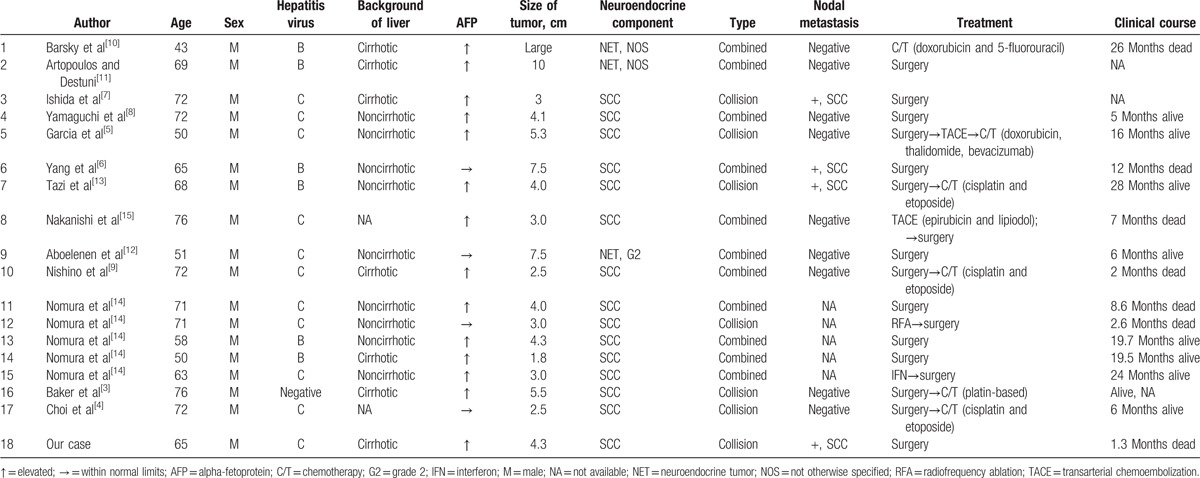
Summary of the clinicopathological profiles of the 18 cases.

These composite tumors were reported as either a collision type or combined type. In the collision tumors, the HCC and NEC grew as microscopically separable compartments. The distinction was grossly discernible in the case described by Garcia et al,^[5]^ in which the HCC component was green and nodular, whereas the SCC component was tan-white and friable. In the combined type of tumors, the HCC and SCC were tightly intermingled and a transition zone could be found. The combined type was more common than the collision type.^[3,6,8–12,14,15]^ In the present case, the 2 components were separated by fibrous septae. Occasionally, poorly differentiated HCC was found juxtaposed to the SCC component. No expression of neuroendocrine markers was found in both well and poorly differentiated HCC. Thus, our case was classified as a collision tumor.

The NEC component was classified as SCC in 15 out of 18 cases.^[3–9,13–15]^ The remaining 3 cases were described as “carcinoid-like.”^[10–12]^ The grading was only available in the case of Aboelenen et al,^[12]^ with the mitotic rate ranging from 2 to 20/10 HPF, which would now be designated as a grade 2 neuroendocrine tumor. None of the 18 cases was functional or had paraneoplastic syndromes. Large cell NEC has so far not been reported in the composite hepatic cancers. These could be explained by scarcity of composite HCC and NEC and overlapping morphology between poorly differentiated HCCs and large cell NEC. Although HCC with NEC component was not mentioned in the 4th edition of WHO Classifications of Tumors of the Digestive System, addressing the morphologic spectrum of the neuroendocrine component might raise the diagnostic rate.

The pathogenesis of the neuroendocrine component is poorly understood. There are 2 hypotheses: the NEC component derives from hepatic stem cells and NEC originates from HCC. Ishida et al^[7]^ described a combined tumor with the HCC component expressing neuron-specific enolase in an immunochemical study and displaying neurosecretory granules under electron microscopy. Yang et al^[6]^ reported a case of a combined type tumor in which poorly differentiated HCC focally expressed neuroendocrine marker CD56. These findings support the postulation that a poorly differentiated HCC dedifferentiates into neuroendocrine phenotype, resulting in a combined HCC with NEC. Recently, Baker et al^[3]^ demonstrated that both HCC and NEC components shared the *CTNNB1* gene mutation (S33F located in exon 3), suggesting that both components might derive from the same cell origin. In our case, the HCC expressed strong β-catenin staining on the membrane, indicating no exon 3 mutation on the *CTNNB1* gene. The SCC showed decreased β-catenin expression on the membrane without cytoplasmic or nuclear staining. The difference in the 2 components indicated divergent underlying molecular events in the composite tumor.

The clinical significance of HCCs with NEC component is unclear.^[5]^ Several studies showed that HCCs with the NEC component are associated with aggressive behavior and a dismal outcome.^[6,8,14,15]^ Reduced expression of β-catenin is reported to associate with tumor dedifferentiation, lymph node involvement, metastasis, and poor prognosis in pulmonary and gastroenteropancreatic neuroendocrine tumors.^[16,17]^ Loss of E-cadherin/β-catenin integrity is described to initiate epithelial-mesenchymal transition, and is generally considered as an early event preceding tumor invasion.^[18]^ In addition to the decreased membranous β-catenin expression, the SCC component in our case was immunoreactive to vimentin with perivascular accentuation. This phenomenon further supported that the SCC component acquired mesenchymal phenotype, leading to early nodal metastasis by this component alone. On the contrary, nodal metastasis is rare in HCC. The incidence ranged from 1.7% to 2.2% in resectable cases.^[19,20]^ The HCC with nodal metastasis are usually infiltrating and larger (tumor size >5 cm).^[19]^ In contrast, 4 of the 18 mixed HCC and NEC have nodal metastasis.^[6,7,13]^ In our case, the primary tumor size was <5 cm, and the lymph nodes harbored SCC rather than HCC. The possibility of a composite component should be considered in small HCCs with nodal metastasis. Clinical awareness of the presence of an SCC component might alter the treatment strategy and eventually the outcome of the patient.

## 6 Conclusion

Mixed tumors of HCC and NEC are still rare. Early diagnosis and adequate treatment requires high clinical suspicion index and then multidisciplinary collaboration.

## Acknowledgments

We would like to specially thank the Tissue Bank and the Department of Pathology at the Chang Gung Memorial Hospital, Guishan, Taoyuan, Taiwan for their excellent tissue processing work.
